# Effects of nutrition and gestational alcohol consumption on fetal growth and development

**DOI:** 10.1093/nutrit/nuab119

**Published:** 2022-05-04

**Authors:** Vishal D Naik, Jehoon Lee, Guoyao Wu, Shannon Washburn, Jayanth Ramadoss

**Affiliations:** Department of Obstetrics & Gynecology, C.S. Mott Center for Human Growth and Development, School of Medicine, Wayne State University, Detroit, Michigan, USA; Department of Veterinary Physiology and Pharmacology, College of Veterinary Medicine and Biomedical Sciences, Texas A&M University, College Station, Texas, USA; Department of Animal Science, Texas A&M University, College Station, Texas, USA; Department of Veterinary Physiology and Pharmacology, College of Veterinary Medicine and Biomedical Sciences, Texas A&M University, College Station, Texas, USA; Department of Obstetrics & Gynecology, C.S. Mott Center for Human Growth and Development, School of Medicine, Wayne State University, Detroit, Michigan, USA; Department of Physiology, School of Medicine, Wayne State University, Detroit, Michigan, USA

## Abstract

Fetal alcohol exposure can lead to a range of developmental disorders, including impaired fetal growth and development of multiple organ systems. These disorders are grouped under the term fetal alcohol spectrum disorders (FASDs). Adequate nutrition and a conducive intrauterine environment are essential for healthy fetal development. Nutrient deficiencies resulting from inadequate maternal nutrient ingestion may be compounded by alcohol-induced altered nutrient metabolism, placental clearance, and malabsorption. Alcohol-induced alteration of the intrauterine environment is the main source of developmental deficits and nutritional insufficiencies can worsen the effects on fetal development. In this review, we discuss studies examining the collective and interactive effects of nutrition (specifically iron, selenium, vitamin A, thiamine, zinc, folate, vitamin B_12_, choline, and amino acids) relative to gestational alcohol consumption and its effects on fetal growth and development. We also summarize scientific reports that tested potential benefits of micronutrient supplementation in animal models of fetal alcohol spectrum disorders and in humans. In summary, the deleterious effects of alcohol exposure in relation to nutrient homeostasis further validate that avoidance of alcohol consumption during pregnancy is the most effective way to mitigate the teratogenic effects of alcohol.

## INTRODUCTION

Fetal alcohol spectrum disorders (FASDs) is a collective term that groups a range of developmental outcomes found in children exposed to all levels of alcohol in utero.^1^ The most severe form of FASD is termed fetal alcohol syndrome (FAS).^2^ The US Surgeon General and the American Academy of Pediatrics have issued an advisory to abstain from any alcohol when considering pregnancy and throughout pregnancy.^3^^,^^4^ The deleterious effect of alcohol during pregnancy is not only environmental; alcohol exerts specific direct effects on molecules and pathways that control fundamental developmental processes.^5^ Although the exact mechanism of FASDs is still elusive, the importance of adequate nutrition during pregnancy for the delivery of healthy offspring has been well documented.^6–9^ Though alcohol is the main source of developmental deficits, nutritional deficits can worsen the teratogenic effect of alcohol on fetal development. Thus, it is equally important to identify the contribution of the nutritional status of the mother before and during pregnancy in addition to gestational, alcohol-induced, secondary downstream effects on the concentrations of the respective nutrients.

Optimal maternal nutrition is essential for maternal well-being and fetal development. It is also a major intrauterine factor in controlling the fetal genome expression and its lifelong consequences. Despite all advancements in prenatal care, approximately 1 in 13 children in the United States has intrauterine growth restriction.^10–15^ Though the intrauterine environment is a major factor in contributing to intrauterine growth restriction, nutrition also plays a critical role in influencing fetal growth.^16^^,^^17^ In addition to the direct deficiencies resulting from a poor dietary intake, alcohol itself can inhibit nutrient metabolism, interfere with placental transfer, induce malabsorption, or lead to decreased nutrient intake. Deficiencies in maternal micronutrients during pregnancy have been associated with adverse outcomes.^18^ For example, a deficiency in iron and zinc can result in fetal growth restriction^19^^,^^20^ by reducing levels of insulin-like growth factor-1 and its receptor activity.^21^^,^^22^ In humans, alcohol consumption was associated with poor nutritional profile when measured against recommended daily allowance or adequate intake by mothers and by children with prenatal alcohol exposure.^23–25^ The interactions of alcohol and nutrients and their effects on the developing fetus are well documented.^26^

Previous review articles in this field have looked at the effects of alcohol on the maternal nutrition profile.^27^^,^^28^ In this review, we examine the effects of gestational alcohol consumption on various micronutrients during pregnancy and their relation to fetal growth and development. Our review presents a detailed analysis of several nutrients including iron, selenium, vitamin A (retinoic acid), vitamin B_1_ (thiamine), zinc, folate, vitamin B_12_, choline, and amino acids in the context of FASDs.

## MATERNAL MICRONUTRIENT AND FETAL DEVELOPMENT DURING ALCOHOL INTERACTIONS

### Iron

**Figure 1:**
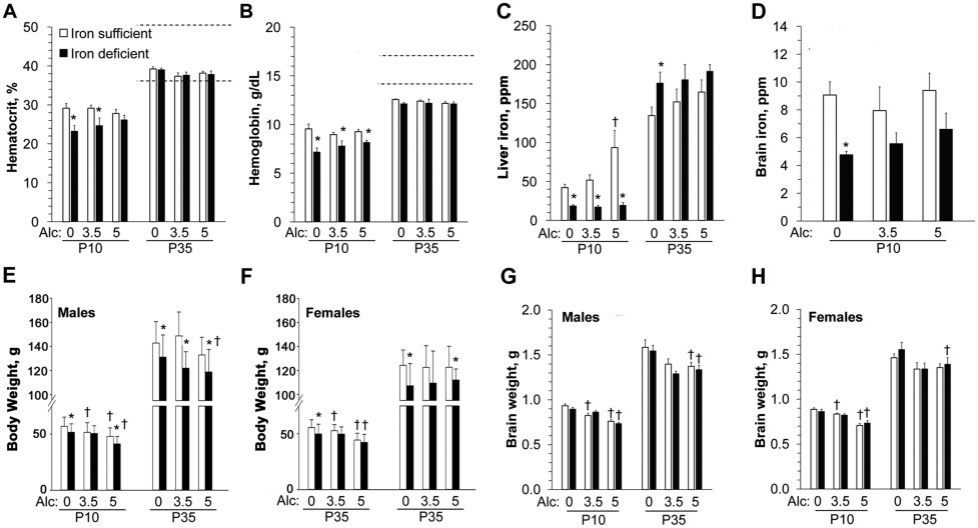
**Reduced iron status and body growth in rat offspring of iron-deficient (ID) dams**.^44^ (A–D) ID offspring are anemic at postnatal day (P) 10, but iron repletion normalizes their iron status by P35. Hematocrit (A), hemoglobin (B), liver iron (C), and brain iron (D) in iron-sufficient (IS) or ID offspring at P10 and/or P35. Dashed lines indicate the normal reference range for nonpregnant adults and do not fully apply to growing animals^45^ (n = 5–13 rats per treatment group at each time point). (E, F) Effect of maternal ID and postnatal alcohol on body weight of P10 and P35 male (E) and female (F) pups treated with indicated alcohol dose (n ≥ 22 rats per treatment group per sex). (G, H) Alcohol significantly diminished male (G) and female (H) whole brain weight on P10 and P35, which was not further altered by iron status (n ≥ 5 rats per treatment group per sex). *Significantly different from age-matched IS pups receiving the same alcohol dose; †significantly different from age-matched animals receiving 0 g/kg alcohol within the same iron status. *Abbreviation:* Alc, alcohol. Adapted from Rufer et al^44^

Maternal iron deficiency concurrent with alcohol consumption during pregnancy has been reported to negatively impact pregnancy outcomes in both animals and humans.^29–31^ Maternal iron deficiency has been reported to result in placental hypertrophy, increased risk of premature delivery, low birth weight, fetal neurodevelopment, and fetal death in studies of animals and humans.^32–35^ The peak blood alcohol concentration after a binge 2.5–7.5 g/kg dose in pregnant rats is ranges from approximately 30 to 525 mg/dL. The higher end of this range has been recorded in women of child-bearing age and those who are admitted to emergency wards.^36–39^ A blood alcohol concentration of 100–400 mg/dL (moderate to binge-like level) produces classical FASD features in rats and is widely used in FASD research with a rat model system.^40^^,^^41^ In humans, moderate to heavy drinking^42^ during pregnancy was associated with an increased incidence of iron-deficiency anemia (assessed at infant ages 6.5 and 12 months), which exacerbated the alcohol effect on infant growth.^30^^,^^43^ Rufer et al^44^ reported that in FASD studies in a rat model, iron deficiency without anemia is a key player in regulating the risk of FASD.^44^ These researchers also reported that binge alcohol exposure (0, 3.5, or 5.0 g ethanol/kg body weight [BW] in milk) in littermate pups from postnatal day (P) 7 to P22 with coexisting iron deficiency diet at gestation day (GD) 5–13.5 (20 ppm) and GD13.5–P7 (4 ppm) impaired somatic growth, associative learning, and white-matter formation during postnatal developments (Figure 1).^44^ In a pregnant rat model, pups with prenatal alcohol exposure (5.0 g ethanol/kg BW) had altered iron distribution (reported in micrograms per gram) in the liver (30% increase in iron-sufficient pups and 60% increase with iron deficiency) and brain (15% decrease in iron-sufficient pups and a 20% decrease with iron deficiency), suggesting altered iron distribution independent of maternal iron levels. An iron-deficient diet worsened the alcohol-associated growth restriction. Interestingly, despite higher iron concentration in the liver, the fetuses were anemic, and an iron-deficient diet worsened the anemia.^46^ A possible explanation for this may involve alcohol’s proinflammatory response resulting in increased hepcidin synthesis affecting iron circulation in fetal liver and brain.

Results of experimental studies of pregnant ewes have shown that alcohol exposure (0.75 g/kg maternal BW; GD95–GD133) during pregnancy decreased fetal liver iron content (mean ± SE: control group, 2.9% ± 0.9%; alcohol group, 0.5% ± 0.2%) and the expression of genes for the iron-regulating hormone hepcidin and tumor necrosis factor-α.^47^ In the placenta, the ferroportin messenger RNA (fold change, mean ± SE: control group, 1.0 ± 0.1; alcohol group, 1.4 ± 0.0) and its protein levels were increased (ferroprotein/actin, mean ± SE: control group, 0.8 ± 0.1; alcohol group, 2.0 ± 0.6) in the alcohol exposure group with no alterations in liver morphology.^47^ In another study, in utero alcohol (2.2% ethanol on GD6–GD7, 4.5% ethanol on GD8–GD10, 6.7% ethanol on GD11–GD21) exposure in rats decreased iron content and increased ferritin concentration in the rat brain, which persisted into adulthood.^48^ Similarly, lower fetal brain iron concentration and increased ferritin concentrations were observed in other rat models.^46^^,^^49^ Iron deficiency and/or alcohol exposure (20% ethanol or 30 ppm iron in the diet) result in decreased maternal red blood cell and serum folate concentration, accompanied by adverse effects on maternal reproductive performance of CBA/J mice.^29^ Alcohol-fed pups (3.5 g/kg/day and 5 g/kg/day in milk, P4–P9) of maternal rats that were fed an iron-deficient diet were anemic, although iron levels were restored in alcohol-fed, iron-deficient pups by the time of weaning. However, the postweaning iron status did not reverse their learning deficits resulting from iron deficiency.^44^

### Selenium

Selenium (Se) is a micronutrient that is essential for maintaining oxidative balance through Se-dependent proteins (eg, glutathione peroxidase).^50–52^ Selenium is associated with fertility in women, and deficiency of selenium during pregnancy can result in dysfunction of the nervous system, such as anencephaly and rachischisis in the developing fetus.^53^ Studies have reported reduced Se levels in plasma and liver and decreased activity of glutathione peroxidase in different organs of men and nonpregnant women with alcohol-use disorder.^54–57^ Maternal drinking during pregnancy showed variable patterns of Se concentrations.^58^ In pregnant women, serum Se levels were decreased with advancing gestational age in women who abstained from and or drank moderate levels of alcohol (42–126 g/week). Study participants who engaged in heavy drinking (>140 g/week) had higher Se levels than those in the abstinent control group between the 25th and 32nd weeks (63.3 ± 6.2 vs 56.6 ± 4.4 µg/L, respectively) and the 33rd to 40th weeks (58.6± 3.4 vs 53.5 ± 5.9 µg/L), respectively.^59^ Studies in rats have shown that alcohol exposure during gestation and lactation (14 weeks, 20% ethanol) is linked to an imbalance in maternal Se homeostasis, causing altered Se retention in tissues, with alcohol-exposed dams retaining more Se in liver, spleen, and heart, and decreased Se in cortex, skeletal muscle, and mammary glands.^60^^,^^61^ In experimental studies on selenium-deficient rats, lower levels of cellular redox status and homocysteine levels have been reported.^62^ Supplementation studies on alcohol-exposed (13 weeks; 5%, 10%, 15%, and 20% ethanol) pups with Se and folate reported preventive effects on the alcohol-induced liver disorder in exposed pups.^63^^,^^64^

### Zinc

Zinc is an essential nutrient required for proteins involved in DNA synthesis, gene transcription, and cell division.^65^ It is crucial for normal growth and development. Maternal zinc deficiency results in an increased rate of abortion, fetal growth restriction, premature birth, and malformation in humans.^66^ Zinc is a cofactor for the alcohol-metabolizing enzyme alcohol dehydrogenase. Zinc deficiency due to ethanol consumption could decrease ethanol metabolism, thus resulting in an increase in ethanol levels in tissues and circulation.^67^ Ethanol ingestion alters zinc metabolism by reducing dietary zinc intake and increasing its excretion in urine.^68^ Infants with FAS had lower plasma zinc levels (control group, 71 ± 1.8 μg/dL; alcohol group, 62.5 ± 2.8 μg/dL) and higher urinary excretion rate of zinc (control group, 76.6 ± 22 μg/24 h; alcohol group, 646 ± 125 μg/24 h) compared with infants born to mothers without an alcohol use disorder.^69^ Another study reported that maternal drinking during pregnancy lowers zinc levels in maternal plasma (control group, 72.2 µg/dL; alcohol group, 50.7 µg/dL) and fetal cord plasma (control group, 81.3 µg/dL; alcohol group, 65.5 µg/dL) compared with plasma of control participants.^70^ Zinc supplementation in humans was associated with increased fetal heart rate and fetal motor activity.^71^^,^^72^

Researchers have also reported similarity of congenital malformations in FAS and zinc deficiency in animals and humans.^68–70^^,^^73^ In experimental studies in rats, alcohol (5%) inhibited the placental transport of zinc to the fetus, thereby inducing fetal growth restriction (placental uptake percentage of injected dose/g: control group, 1.37 ± 0.02, alcohol group, 0.86 ± 0.02; fetal uptake percentage of injected dose/g: control group, 0.09 ± 0.01, alcohol group, 0.05 ± 0.002).^73–75^ Others have reported that mice fed a zinc-deficient diet (8.5 µg/mL) and alcohol (0%, 15%, or 20%) exhibited a teratogenic effect of alcohol influenced by zinc deficiency.^75^^,^^76^

In studies of zinc supplementation, zinc (5, 10, 40 mg/L) did not reverse the effect of ethanol (5%) on placental transport of zinc and fetal cerebral development.^73^^,^^77^ Fetuses from pregnant mice fed low doses of alcohol (2 µg/g of 50% ethanol) and zinc (10 µg/g) for 18 days of pregnancy had increased external defects, including decreased fetal weight, hematoma, and maxillary defects; and internal defects, including hydronephrotic kidney, involuted thymus, domed heads, necrotic lung and liver, hemorrhagic noses, cleft palate, necrotic bowel, and hemorrhagic diaphragm.^78^ Excess alcohol intake has been reported to increase lipopolysaccharide levels in the digestive tract of humans and animals, and zinc supplementation alleviated lipopolysaccharide-induced preterm delivery, fetal death, fetal skeletal development retardation, and fetal growth restriction in pregnant mice.^79^ In utero exposure of alcohol (25% ethanol) in pregnant mice resulted in impaired spatial memory, whereas zinc supplementation (GD8; 2.5 µg zinc/g) given at the time of alcohol exposure confined spatial memory impairments, ameliorated dysmorphology and postnatal mortality rate (mean ± SEM: control group, 20 ± 0.27; alcohol group, –6.38 ± 0.27; alcohol + zinc group, −5.06 ± 0.29).^80^^,^^81^ Alcohol (4.5 g/kg/day; PD4–9) exposure in rat pups during the brain development period resulted in cerebellar Purkinje cell loss, and zinc supplementation (0.54 mg/mL diet) did not rescue alcohol-induced developmental cerebellar Purkinje cell loss.^82^

### Vitamin A

Vitamin A is essential for the differentiation of fertilized eggs into different organs of the body. Retinoic acid, a bioactive form of vitamin A, plays a major role in development. Vitamin A deficiency is reported to substantially alter placental development and pregnancy outcome, affecting embryonic development and resulting in ocular defects in rodent offspring and, in some cases, fetal death.^83–85^ Consumption of alcohol has a negative effect on vitamin A metabolism,^86^^,^^87^ which is not yet completely understood.

Alcohol consumption in humans resulted in vitamin A deficiency, according to study findings.^88^ It is also reported that chronic alcohol consumption in humans and in nonpregnant animals results in the depletion of vitamin A stores in the liver.^89^^,^^90^ From a case study, researchers reported that prenatal alcohol exposure (2 bottles/day) throughout pregnancy resulted in vitamin A deficiency and developmental defects in the brain of a newborn.^91^ Similarly, in a study examining factors influencing vitamin A deficiency in pregnant Chinese women (factoring in city size, economic status, gestational age at time of blood collection, use of a vitamin A supplement, and if the women drank or smoked during pregnancy), researchers reported that those who consumed alcohol during the past 12 months were 3 times more likely to have vitamin A deficiency than were pregnant women who did not drink alcohol.^92^ Maternal ingestion of alcohol (36%) in pregnant rats from GD4 to GD21 resulted in lower liver palmitate levels (nmol/g ± SEM: control group, 43.43 ± 2.10; alcohol group, 28.53 ± 2.38) and total retinyl palmitate (nmol/g ± SEM: control group, 11.40 ± 0.41; alcohol group, 5.81 ± 0.39) in the liver, kidney, and lungs of fetuses on GD21.^93^ In another study, researchers reported that ingestion of 36% alcohol by pregnant rats from GD1–GD20 resulted in increased retinol levels (nmol/g ± SEM: control group, 0.46 ± 0.059; alcohol group, 1.06 ± 0.175; *P* < 0.01) and retinyl palmitate levels (nmol/g ± SEM: control group, 0.71 ±0.09; alcohol group, 1.59 ± 0.24; *P*  < 0.01) and lower levels of retinoic acid (nmol/g ± SEM: control group, 2.49 ± 0.27; alcohol group, 1.55 ± 0.18; *P* < 0.02) in fetal hearts.^94^

It is suggested that ethanol-induced FAS results partly from a reduced availability of retinoic acid.^95^^,^^96^ Supplementation studies of retinoic acid (50 or 100 µg/kg BW) could restore retinoic acid levels in the liver and reduce some type of ethanol-induced liver injury in adult male rats.^97–99^ Retinoic acid supplementation in zebrafish embryo gastrulation and somitogenesis stages also partially reversed some of the dysmorphic changes associated with FASD.^86^

### Thiamine (vitamin B_1_)

Thiamine (vitamin B_1_) is an important water-soluble B vitamin required for a successful pregnancy, and it influences reproductive functions as well as fetal development.^100–102^ Thiamine deficiency results in adverse effects on fetal growth and brain development in rat models, with and without alcohol use.^100^^,^^103^^,^^104^ Thiamine is transferred from the mother to the fetus. Thiamine status and metabolism are altered by excessive alcohol intake in 3 ways: (1) increasing the demands for thiamine for the catabolism of alcohol, (2) impaired absorption of the vitamin by the small intestine due to inhibition of intestinal ATPase, and (3) displacement of daily energy intake by alcoholic beverages.^105^ In a case study, 20 infant children (n = 12 boys and 8 girls) who had thiamine-deficient diets were reported to have delayed language development in their childhood.^106^ Severe thiamine deficiency occurs in alcoholism and can lead to Wernicke-Korsakov syndrome.^107^ In experimental studies of rats, the coexistence of alcohol consumption (12%) and thiamine (0.2 g/L) deficiency during pregnancy resulted in fetal death (control group, 84.47%; alcohol group, 48.26%), reduced fetal size (control group, 72.7%; alcohol group, 44.54%), and lower birth weights (control group, 5.5 ± 0.22 g; alcohol group, 2.9 ± 0.16 g).^108^ Supplementing dams with a thiamine-enriched diet reversed the effect of alcohol-induced fetal deaths; however, supplementation only partially reversed fetal weight and did not affect litter size.^108^ Alcohol-thiamine antagonism primarily affects cellular differentiation and it peaks during the perinatal stage in rats.^109^ Another study reported that rats fed only a thiamine-deficient diet (< 0.08 mg/kg) had more profound effects on fertility and fetal development than did a high alcohol diet. Researchers have also reported that chronic alcohol (12%) administration to rats throughout gestation and during lactation influences alterations in thiamine metabolism, resulting in fetal neurobehavioral developmental disorder and reduced hippocampal CA3 pyramidal cell number and size.^110^ Thiamine administration improved alcohol-induced behavioral effects such as habituation, emotional reaction, and neurodevelopmental defects such as soma width in the hippocampal CA3 in offspring, but did not improve cell number or soma length.^110^

### Folate and vitamin B_12_

Folate and vitamin B_12_ are required for DNA synthesis and cell proliferation during pregnancy. A major function of folate is remethylation of plasma homocysteine to methionine. Folate deficiency during pregnancy can affect methylation of DNA and can lead to neuronal tube defects due to hyperhomocystenemia.^111^ Fetal levels of folate are 2- to 4-fold higher than maternal levels.^112–114^ Alcohol is reported to induce oxidative stress in rat placenta,^115^ and folate has been reported to alleviates oxidative stress^116^ Chronic alcohol consumption resulted in folate deficiency and malabsorption of vitamin B_12_.^117–120^ Researchers have reported decreased transport of folate (control group, 5.91 ± 20.73; alcohol group, 33.15 ± 19.89) from the mother to fetus during gestational alcohol consumption.^121^ In another study, researchers reported altered folate and vitamin B_12_ metabolism in people with chronic alcohol use disorder and heavy drinkers (n = 31; folate: control group, 162.7 ± 54.5, alcohol group, 128.7 ± 56.8; vitamin B_12_: control group, 221.0 ± 80.6, alcohol group, 398.1 ± 347.8).^122^ Experimental studies have revealed impaired folate transport due to alcohol exposure between the maternal and fetal compartments, resulting from decreased expression of folate transport proteins.^123–126^

Folate supplementation has a range of beneficial roles in preventing birth defects, specifically neurologic abnormalities.^127–129^ In experiments, supplementation of folic acid (60 mg/kg) and vitamin B_12_ (1.0 mg/kg) mitigated developmental toxicity induced by alcohol (5.0 g/kg) in mice.^130^ Researchers using a zebrafish model reported folic acid supplementation (75 µM) reversed alcohol-induced (100 mM and 150 mM ethanol, 2–24 h postfertilization) disruption of retinal morphogenesis, specifically optic nerve and photoreceptor differentiation defects.^131^ In another study, researchers reported that folic acid (60 mg/kg) supplementation reversed fetal brain weight deficits (brain weight: control group, 447.71 ± 16.91; FAS, 398.83 ± 28.08; alcohol + folic acid, 427.90 ± 19.05) and BW deficiency (BW: control group, 1.38 ± 0.14; FAS, 1.30 ± 0.12; alcohol + folic acid, 1.34 ± 0.09) in alcohol-fed pregnant mice.^132^

### Choline

Choline is an essential nutrient and a precursor for the neurotransmitter acetylcholine and other cellular constituents like phosphatidylcholine and sphingomyelin.^133^ It also serves as a methyl donor by influencing DNA methylation and gene expression regulation. Choline is essential during pregnancy for fetal development, and its deficiencies can result in neural tube defects and central nervous system dysfunction.^134^ Maternal alcohol consumption during pregnancy induces a deficiency of choline, resulting in neurobehavioral defects possibly mediated by changes in neurotransmitter levels, DNA methylation alterations, and gene expression–pattern differences.^135^

**Figure 2:**
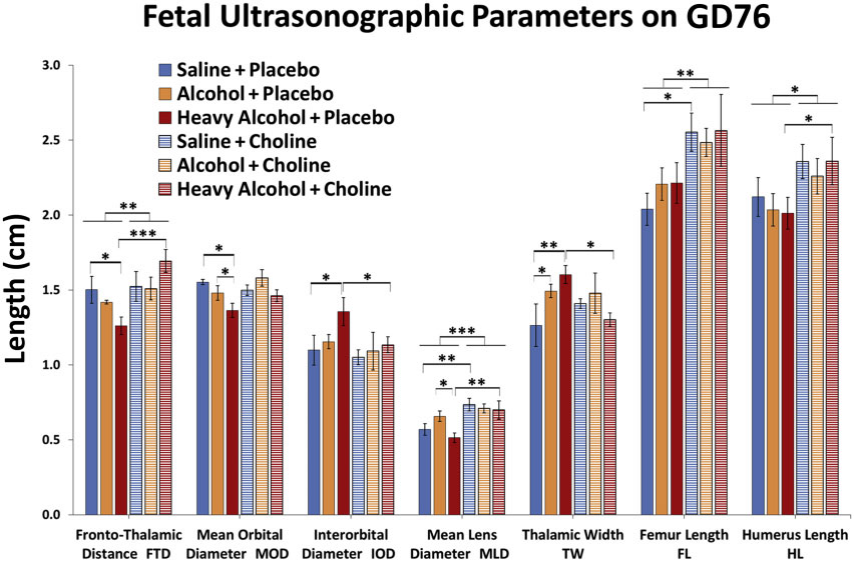
**Ovine fetal ultrasonographic parameters on gestational day (GD) 76**.^145^ Values are reported as mean ± SEM. *P < 0.05; **P < 0.01; ***P < 0.001. Adapted with permission from Sawant et al^145^

Studies of children with FAS revealed that maternal alcohol consumption during pregnancy lowered choline concentrations in the frontal lobes of the fetal brain.^136^^,^^137^ Experimental studies in rat fetuses found that alcohol exposure (36%) decreased hippocampal neurogenesis and cell survival.^138–140^ In addition, decreased choline availability to the fetus also decreases hippocampal neurogenesis and enhanced neuronal apoptosis.^141^ Maternal supplementation of choline (250 mg/kg) mitigates adverse effects of prenatal alcohol exposure (6 g/kg; GD5–GD20) in the rat during development.^137^^,^^142^ In humans, infants whose mothers drank heavily during pregnancy, who received choline supplementation (2 g/day until delivery) showed considerable catch-up growth weight, head circumference, and higher novelty preference score at 12 months postdelivery, compared with the placebo group.^143^ In another study, choline (100 mg/kg/day) supplementation in rats fed 5.2 g/kg/day ethanol attenuated learning deficits.^144^ In sheep, choline supplementation mitigated some of the alcohol-induced fetal craniofacial abnormalities (Figure 2).^145^^,^^146^

### Amino acids in FASD

Maternal alcohol consumption during pregnancy interferes with folate and amino acid metabolism^147–149^ and induces nutrient deficiency, thus resulting in fetal growth and impairments.^150^ Fetal growth and development demand a continuous supply of amino acids from the mother to the fetus. Amino acids cross the placenta and some amino acids (eg, glutamine and alanine) are synthesized by this organ, giving rise to the high fetal–maternal ratio of certain amino acids.^151–153^ It has been demonstrated that a number of amino acids are reduced in both maternal and fetal compartments in response to gestational alcohol exposure.

Studies of 6 children with FAS born to mothers with alcoholism showed there were substantial changes in serum amino acid patterns (ie, a decrease in hydroxyproline and proline and an increase in alanine, leucine, isoleucine, and tyrosine levels) indicating skeletal and central nervous system developmental disorder.^154^ Acute and chronic alcohol administration studies in pregnant mice and rats revealed that alcohol markedly decreased the plasma concentration of amino acids such as threonine, serine, glutamine, glycine, alanine, methionine, and proline.^155–157^

Glutamine, one of the most abundant amino acids in fetal plasma, plays an important role in whole-body nitrogen metabolism.^158^^,^^159^ Alterations in glutamine homeostasis could result in alterations in the glutamine-dependent synthesis of some amino acids, such as arginine and citrulline. In 2 case studies, infants born with mutations in the glutamine synthase gene had brain malformation, severe enteropathy, and necrolytic erythema of the skin.^160^ Chronic binge alcohol exposure (1.75 g/kg) in pregnant ewes with the blood alcohol concentration 260 mg/dL induced acidosis, thus resulting in decreased levels of glutamine (control group, 179 ± 16; alcohol group, 112 ± 15) and other amino acids in maternal plasma circulation. Data from clinical studies indicate chronic alcohol-induced mixed respiratory, metabolic acidosis, and alterations in blood pH of patients with alcoholism.^161–164^ Experimental studies in FASD animal models revealed the changes in arterial partial pressure of CO_2_ and arterial pH from prenatal alcohol consumption.^165–168^ Alterations in pH affected glutamine/glutamate metabolism and the transport of amino acids across the cell membranes.^169^^,^^170^ According to results of supplementation studies in pregnant ewes, acute administration of glutamine (30 and 100 mg/kg) concurrent with alcohol consumption (1.75 g/kg) improved maternal and fetal plasma amino acid profiles, alleviated alcohol-induced fetal growth restriction, and suppressed alcohol-induced mTOR signaling in fetal skeletal muscle.^171–173^

**Figure 3:**
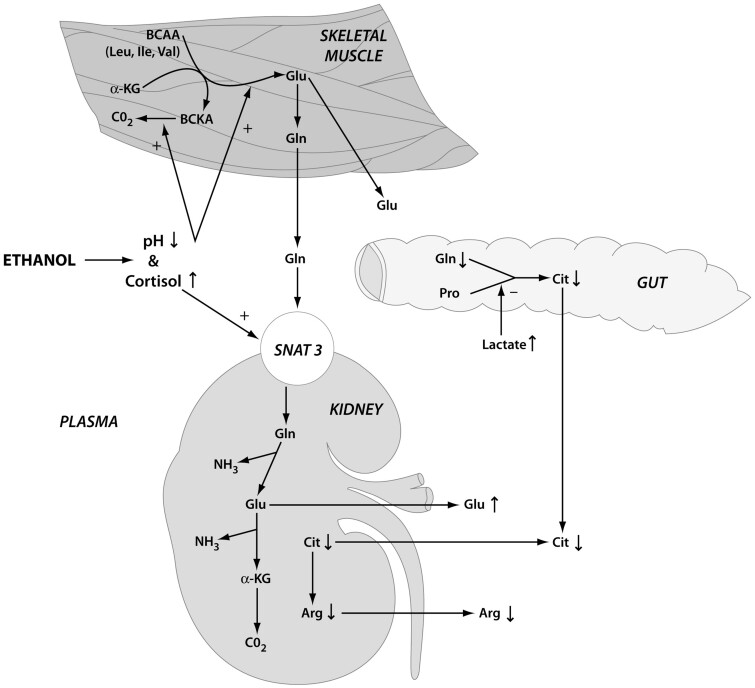
**The ethanol–glutamine model illustrating putative maternal amino acid homeostatic responses to an acute challenge of ethanol after chronic exposure**.^181^ The kidney acts as a major sink for glutamine during ethanol-induced acidosis. Increased renal extraction of plasma glutamine results in decreased availability of arginine and citrulline, whose syntheses are glutamine dependent. Acidemia-induced elevations in renal glutamine SNAT3 transporter expression result in increased renal uptake of histidine and asparagine. In the muscle, ethanol-induced acidosis upregulates the transamination of branched-chain amino acids (BCAAs) with α-ketoglutarate to form glutamate and branched-chain α-ketoacids (BCKAs) and also directly stimulates the oxidative catabolism of BCKAs, leading to decreased plasma levels of BCAAs. Adapted with permission from Ramadoss et al^181^

Methionine is a nutritionally essential amino acid in humans^174^ and is a precursor for glutathione synthesis.^175^ Ethanol-induced restriction in availability and absorption of maternal methionine could affect the developing fetus. Ethanol interferes with the intestinal transport of methionine in humans and rats.^176^^,^^177^ Experimental studies revealed that chronic ethanol (40% of kilocalories) fed to micro pigs for 12 months reduced serum methionine levels.^178^ Similarly, in pregnant mice, ethanol (25% volume per volume) induced numerous neural and physical malformations, accompanied by a reduction in several amino acids, including methionine.^155^ Pretreatment with a single dose of methionine (70 mg/kg) in alcohol-treated pregnant mice on 1 day between GD7 and GD12 improved only certain alcohol-induced non-neural malformations of cleft palate and limbs.^155^ In rats, 14 days of alcohol administration (2.5 g/kg and 5 g/kg) significantly increased blood methionine levels (control group, 0.206 ± 0.037 mg/dL; 2.5 g/kg ethanol group, 0.246 ± 0.030 mg/dL; 5 g/kg ethanol group, 0.271 ± 0.059 mg/dL).^179^ An experimental study of female pregnant rats revealed that alcohol exposure (4.3% weight per volume; GD6–GD20 or GD21) resulted in a decrease in the concentrations of many amino acids in fetal plasma, but methionine concentration did not change.^180^ Exposure to chronic binge alcohol levels (1.75 g/kg; GD109–GD132) in pregnant ewes increased maternal plasma methionine levels compared with levels in the control animals (control group, 13 ± 0.5 µmol/L; alcohol group, 20 ± 2 µmol/L) as a physiological adaptation (Figure 3).^181^

l-Leucine, a nutritionally essential branched-chain amino acid, functions as a nutrient regulator of protein synthesis in skeletal muscle and an activator of messenger RNA translation.^182^^,^^183^ The uptake of leucine by tissues is an active transport process.^184–187^ Ethanol interferes with leucine transport across cell membranes by interacting with the hydrophobic region of the membrane.^188^ Experimental studies revealed that ethanol (200 mg/dL) interferes with amino acid transport in the rat fetal brain.^189^ In another study, maternal exposure of ethanol (47.5%) at a high dose (0.33 g/100 g BW) affected leucine uptake (control group, 1879 ± l85 dpm/mg protein; alcohol group, 1219 ± 123 dpm/mg protein) and altered fetal metabolism.^190^ Other researchers have found that chronic binge alcohol exposure (1.75 g/kg; GD109–132) in pregnant ewes resulted in increased maternal plasma leucine levels (control group, 66 ± 7 µmol/L; alcohol group, 84 ± 7 µmol/L) compared with control groups, possibly due to an inhibition of leucine catabolism.^181^ Supplementation studies in the FASD zebrafish model revealed that 50 mM l-leucine supplementation along with ethanol (1.0%) in zebrafish embryos partially inhibited ethanol-induced craniofacial defects.^191^

Histidine is a nutritionally essential amino acid required for fetal development.^192^ Alcohol consumption during pregnancy interferes with histidine transport, resulting in reduced maternal absorption or reduced placental transfer.^193^ The concentration of free histidine in plasma is highly influenced by ethanol intake.^180^^,^^194^ An experimental study revealed that free amino acids in plasma from ethanol-treated fetuses (4.3% weight per volume; GD6–GD20 or GD21) were lower than in control fetuses; specifically, histidine levels were significantly lower in the alcohol group than in the control (control group, 82.1 ± 4.4; alcohol group, 48.6 ± 4.8; 41% lower). Similarly, fetal plasma histidine levels were significantly lower than those in controls (control group, 105.4 ± 11.3; alcohol group, 1.6 ± 7.0; 51% lower) and, likewise, the fetal–maternal plasma ratio for histidine was lower (control group, 2.96; alcohol group, 1.31; 56% lower) in the alcohol group than in the control group.^180^ In another study, the same researchers reported that the effect of alcohol (35%; GD7–GD21) differs among different tissues. Amino acids in maternal tissue were more consistent between control and alcohol-fed groups, whereas in the alcohol group compared with the control group, several of the fetal tissues consistently showed a decrease in histidine levels, including the fetal plasma (control group, 85.3 ± 4.5; alcohol group, 51.8 ± 6.0), liver (control group, 503.7 ± 47.3; alcohol group, 269.0 ± 26.4), and brain (control group, 154.6 ± 8.7; alcohol group, 117.9 ± 7.7). Furthermore, the distribution of radiolabeled histidine (^14^C-histidine) in the fetal blood was significantly decreased (control group, 0.369 ± 0.016; alcohol group, 0.336 ± 0.036) in the alcohol-fed group than in the control group.^194^ Histidine supplementation (3 weeks; 0.5, 1, and 2 g/L in drinking water) in an FASD mouse model alleviated alcohol-induced chronic liver injury by inducing dose-dependent antioxidative and anti-inflammatory effects.^195^ It is possible that hepatic dysfunction impairs the catabolism of histidine, leading to an elevation of its concentration in the plasma.^196^

## CONCLUSION

In this review, we summarized scientific reports of testing potential benefits of micronutrient supplementation in both FASD animal models and humans.^81^^,^^110^^,^^130^^,^^198^^,^^199^^,^^200^ The deleterious effects of alcohol consumption during pregnancy and its interaction with maternal micronutrient status can markedly affect fetal development. Although nutrient supplementation has not been shown to completely reverse FASD, abstaining from alcohol use during pregnancy remains the main preventive intervention. Furthermore, socioeconomic factors, level of education, age, and ethnicity are not the only determining factors for FASD vulnerability; thus, increasing awareness through education, media, and governmental interventions must remain the main focus to further spread awareness of FASD.^197^ In conclusion, alcohol’s deleterious effect on maternal and fetal nutritional homeostasis further supports abstinence from alcohol during pregnancy as the best preventive strategy for FASD.
